# How our authors are using AI tools in manuscript writing

**DOI:** 10.1016/j.patter.2024.101075

**Published:** 2024-10-11

**Authors:** Yinqi Bai, Clayton W. Kosonocky, James Z. Wang

**Affiliations:** 1 BGI-Research, Sanya 572025, China; 2 Department of Molecular Biosciences, University of Texas at Austin, Austin, TX 78705, USA; 3 Data Sciences and Artificial Intelligence Area, College of Information Sciences and Technology, The Pennsylvania State University, University Park, PA 16803, USA

## Abstract

Scientific writing is an essential skill for researchers to publish their work in respected peer-reviewed journals. While using AI-assisted tools can help researchers with spelling checks, grammar corrections, and even rephrasing of paragraphs to improve the language and meet journal standards, unethical use of these tools may raise research integrity concerns during this process. In this piece, three *Patterns* authors share their thoughts on three questions: how do you use AI tools ethically during manuscript writing? What benefits and risks do you believe AI tools will bring to scientific writing? Do you have any recommendations for better policies regulating AI tools’ use in scientific writing?

## Main text

### Yinqi Bai

**Figure:**
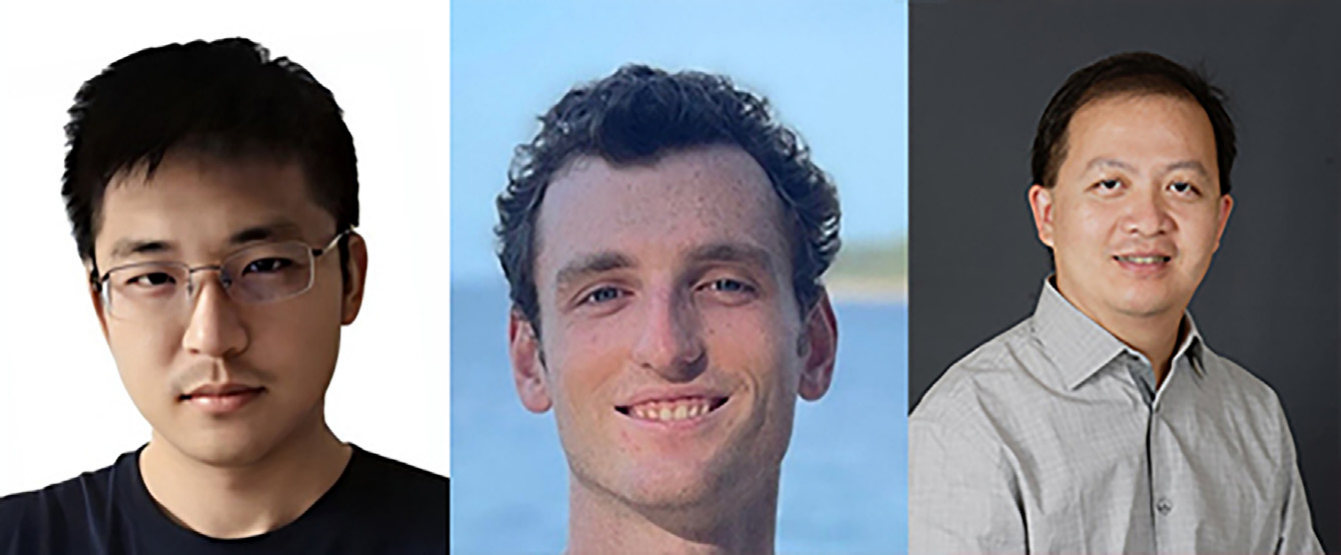
From left to right: Yinqi Bai, Clayton W. Kosonocky, and James Z. Wang.

As an algorithm developer, I frequently use ChatGPT-4 during manuscript writing. There are two major applications: the first is to explore the functions of a gene that shows statistical significance in differential gene expression analysis. After obtaining keywords related to the target gene, I use Google Scholar for broader knowledge collection. The second, more common, scenario occurs when I write a paragraph or long sentences. In these cases, I usually ask ChatGPT to “proofread the sentences of an academic paper.” It makes minor adjustments to enhance the fluency and clarity of my expression.

I am not a native English speaker, and although I have practiced extensively, I believe my written English may not yet be comparable to that of native speakers without the assistance of AI tools such as ChatGPT. By utilizing these tools for language proofreading, I can focus more on the research itself rather than on how to articulate my findings. However, the misuse of AI tools—such as asking them to generate research conclusions or to cite fabricated references—could significantly mislead both reviewers and readers.

Currently, my lab is focused on developing an AI robot to facilitate autonomous bioinformatic analysis based on the methods sections provided in research articles. We hope this tool will assist journals in quickly detecting the extent of AI involvement in scientific work. I believe it is crucial to promote transparency by clearly distinguishing between the use of AI tools for language proofreading and their use in data or result fabrication.

### Clayton W. Kosonocky

During the writing of our manuscript, we used ChatGPT quite frequently to rephrase clunky sentences.^1^ To do this, we would provide our sentence and ask it to clarify and simplify the language. In almost every case, this made the sentence more readable and helped improve the overall flow of our paper. Since we were providing all necessary factual information, it made it easier for the model to get things right when rewriting our words. But even so, we still reviewed all generated text and made further human edits to keep our style. We did not use it to generate entire sentences *de novo*, as this tended not to work given the highly specific topic we were writing about.

AI has the potential to greatly increase the clarity of scientific writing and speed of manuscript creation. However, these models can greatly pollute scientific thought if used improperly. AI models should not be blindly trusted to understand and generate sentences about your research, as research should be inherently novel and thus out of distribution for the model.

Regulating an immature technology can result in unintended consequences. To enable maximal benefit at this time, it seems reasonable to communicate that the burden of responsibility should rest on the authors that use these tools.

### James Z. Wang

When it comes to writing for publication, I have always been quite particular: it often takes me multiple iterations of carefully editing each word and sentence. As a non-native speaker, this process is critical to me, as it ensures the clarity, precision, and impact of my writing. Over time, as my career has advanced and the number of concurrent projects and responsibilities in mentoring doctoral students has increased, I have stayed deeply involved in every stage of the manuscript development process, from drafting to final editing. I have integrated AI tools into my workflow to make this rigorous process more efficient while maintaining the same high level of quality.

Our data science research is driven by the collaborative efforts of computing scientists, statisticians, and domain experts from various disciplines. The initial drafting of manuscripts is handled by doctoral students, most of whom are non-native speakers, and faculty members, without the use of AI tools. In the manuscript finalization stage, I work closely with other authors to refine the scientific arguments and enhance the clarity of the writing. Previously, I might have spent considerable time refining a single sentence to improve its structure, logic, grammar, and word choice. With the introduction of ChatGPT-4, I have found this tool particularly useful for low-level editing tasks, such as correcting grammar, rephrasing awkward sentences, and adjusting word choice, and it has assisted in our recent papers published in *Patterns*.^2^^,^^3^

I can input a draft paragraph into ChatGPT and request revisions as needed. I personally compare its suggestions with the original text to determine whether each change (1) enhances the language quality or improves reader comprehension, (2) preserves the nuance of the scientific argument, and (3) retains the personal tone of the writing. Only suggestions meeting all three criteria are implemented. When reviewing text written by a highly experienced colleague—especially in sections introducing the domain problems we are addressing with data science approaches—I use tools like Grammarly to catch any minor typographical or grammatical errors that might have been overlooked, as the likelihood of ChatGPT enhancing the writing quality in these cases is minimal. After all edits, the manuscript undergoes a final review by a coauthor and/or a professional copy editor to ensure the highest standards of clarity and precision.

Beyond editing, I also leverage ChatGPT for technical tasks in writing, such as formatting assistance. For example, if I need a specific LaTeX command to adjust the appearance of a figure with arrays of subfigures, I may ask ChatGPT to refine our LaTeX codes to achieve the desired layout. While its responses aren’t always accurate, they provide a useful starting point, saving me time before I conduct more in-depth web searches.

Finally, I use ChatGPT as a brainstorming partner for design aspects. When creating a figure to convey a specific idea or concept, I might seek suggestions from ChatGPT on how to structure the illustration or what color palette to use. Although many of its ideas may not be directly applicable, they often help broaden my perspective and explore different approaches to figure design.

AI tools offer several unique advantages. They are available 24/7, providing immediate feedback whenever needed. Unlike typical copy editors who may specialize in language and communication, AI tools can integrate writing-related knowledge from technical disciplines directly relevant to the manuscript, which is particularly useful for interdisciplinary topics. Additionally, they can suggest alternative phrasings to better fit the desired tone and help identify and address coherence and flow issues by considering the broader context of the writing. By interacting with AI tools through natural language, authors can also improve various aspects of their writing, including grammar, vocabulary, conciseness, and syntax.

In the long run, as the language quality of manuscripts reaches a sufficiently high standard with the help of AI tools, reviewers will be able to focus more on the novelty of the ideas, the methodological rigor, the potential impact of the work, and the overall structure and clarity of the writing. This shift, in my view, is healthy for the scientific community, as it encourages authors to prioritize these critical dimensions in their efforts to publish in the most impactful venues.

However, as with any new technology, the benefits of AI tools come with profound risks. One concern is that if researchers rely on AI tools for *drafting* content, there is no guarantee of originality, accuracy, or creativity. AI models inherently reflect the biases present in their training data, which may prevent them from meeting the rigorous standards required for scientific publishing. Additionally, as early-career researchers (e.g., graduate students) become dependent on AI to polish their manuscripts, they may invest less effort in developing their writing skills. This could impair their ability to articulate complex ideas, much like how relying on GPS for navigation can diminish one’s ability to read maps, ultimately reducing one’s sense of direction and spatial awareness. Furthermore, AI-generated text often sounds “robotic”—mechanical, unnatural, and lifeless—so overreliance on AI can diminish the writer’s unique voice: the distinct style, tone, and insights that make scientific writing more engaging and impactful.

A long-term risk, however, isn’t that AI will produce robotic text but rather that it will create text so compelling, so logically flawless, and so adaptable to an author’s style that human authorship may be marginalized. While this may seem distant, most of us wouldn’t have expected to see a tool like ChatGPT in our lifetime, either. The just-released ChatGPT o1, with its incorporation of logical reasoning capabilities, is already a significant step in this direction.^4^ I believe the scientific community must decide—if not now, then soon—whether it will resist or embrace this revolution.

Given their limitations, I believe current AI tools can enhance writing quality for experienced authors, especially those who are time constrained and in fields such as natural sciences, engineering, and interdisciplinary sciences. Inexperienced authors in particular need to be more aware of these limitations and exercise caution. To keep the diversity of perspectives in scientific discourse alive, I believe we should develop AI tools that can clearly explain how they make decisions. This way, authors can engage with AI’s suggestions, learning from them as they would from a helpful colleague.

When a powerful tool like ChatGPT is available, it is neither beneficial nor feasible to prevent its use. However, the scientific community must remain vigilant about the ethical implications and long-term impacts. My former mentor, American number theorist Dennis A. Hejhal (born 1948), who pioneered the use of supercomputing to study deep mathematical structures, remarked in the late 1990s about the Internet, “It pays to have a healthy skepticism about the miracles of modern technology.”^5^ This sentiment has stayed with me throughout my career, and in a recent conversation, he quipped that he hasn’t changed his view one iota. His perspective is particularly relevant as we navigate the integration of AI tools into scientific writing.

As essential contributors to maintaining the integrity of scientific communication, publishers could take a more proactive role by establishing clear guidelines that encourage a model of author-AI collaboration that promotes AI tools as supplements to, rather than replacements for, the critical thinking and creativity of authors. Publishers should require authors to clearly disclose the use of AI tools and prohibit their use for generating original content. Authors should also certify that they have thoroughly reviewed and edited the content as necessary and take full responsibility for its accuracy and integrity. To facilitate transparency, publications could designate a standardized section in manuscripts for reporting AI tool usage. Additionally, publishers could promote the open sharing of best practices and emerging risks within the scientific community. Finally, regularly reviewing and updating policies is essential to keep pace with technological developments.

### Elsevier policies

We require that authors acknowledge the use of any AI-based tools during the writing or editing process. Please see “The use of generative AI and AI-assisted technologies in writing for Elsevier” for more information on our policies with regard to AI writing tools.

## Acknowledgments

J.Z.W.’s current research is primarily funded by the US National Science Foundation (NSF), the National Institutes of Health, the National Endowment for the Humanities, and the Burroughs Wellcome Fund. Any opinions, findings, and conclusions or recommendations expressed in his contributions are solely his and do not necessarily reflect the views of these agencies.

## Declaration of interests

The authors declare no competing interests.

## Declaration of generative AI and AI-assisted technologies in the writing process

During the preparation of his response, J.Z.W. used ChatGPT-4o to improve readability and language. After using this tool, he reviewed and edited the content as needed and takes full responsibility for the final publication.

Y.B. acknowledges the assistance of ChatGPT-4 for providing proofreading support during the writing process.

## References

[1] Kosonocky C.W., Feller A., Wilke C.O., Ellington A.D. (2023). Using alternative SMILES representations to identify novel functional analogues in chemical similarity vector searches. Patterns.

[2] Wu C., Davaasuren D., Shafir T., Tsachor R., Wang J.Z. (2023). Bodily expressed emotion understanding through integrating Laban movement analysis. Patterns.

[3] Zhu L., Wang J.Z., Lee W., Wyble B. (2024). Incorporating simulated spatial context information improves the effectiveness of contrastive learning models. Patterns.

[4] Collins B. (2024). New ChatGPT o1 Blitzes Britain’s Hardest TV Quiz. https://www.forbes.com/sites/barrycollins/2024/09/13/new-chatgpt-o1-blitzes-britains-hardest-tv-quiz/.

[5] Wang J.Z. (2001). Integrated Region-Based Image Retrieval.

